# EXAMINING RELIGIOUS DOUBT AND WELL-BEING OVER TIME

**DOI:** 10.1093/geroni/igz038.1303

**Published:** 2019-11-08

**Authors:** Julie Hicks Patrick, Laura Bernstein, Madeline M Marello, Heather R Moore

**Affiliations:** West Virginia University, Morgantown, West Virginia, United States

## Abstract

Drawing on seminal pieces in the field (e.g., Krause et al., 1999; Krause & Ellison, 2009) and our own work (e.g., Henrie & Patrick, 2014; Patrick & Henrie, 2015; 2016), we examine several aspects of religious doubt, including: 1) identifying a multidimensional measure, 2) replicating cotemporaneous associations among doubt and key well-being outcomes, 3) demonstrating the longitudinal stability of doubt over a 2-year period, and 4) examining whether doubt influences the stability or levels of well-being over a two-year period. Our factor analysis supports a 4-factor model of religious doubt, with stability over the 2-year period. Associations among religious doubt and well-being were consistent across multiple samples. Latent growth curve models showed that religious doubt and age significantly influence mean levels of well-being, although neither age nor doubt influenced the trajectory of change in well-being over the 2-year period. Future directions for design and analyses are discussed.

